# Microangiopathy—A Potential Contributing Factor to Idiopathic Polyneuropathy: A Mini Review

**DOI:** 10.3389/fneur.2018.00043

**Published:** 2018-02-12

**Authors:** Kristin Samuelsson, Rayomand Press

**Affiliations:** ^1^Department of Clinical Neuroscience, Karolinska Institutet, Solna, Sweden; ^2^Department of Neurology, Karolinska University Hospital, Stockholm, Sweden

**Keywords:** idiopathic, microangiopathy, polyneuropathy, sural nerve biopsy, vasa nervorum

## Abstract

Chronic idiopathic axonal polyneuropathy (CIAP) is a slowly progressive predominantly sensory axonal polyneuropathy. The prevalence of CIAP increases with age. The pathogenic cause of CIAP is unknown although there are several prevailing etiological hypotheses. In this mini review, we focus on the hypothesis of disturbed microcirculation in the vasa nervorum of peripheral nerves as a pathogenic cause of CIAP. There is an association between CIAP and metabolic risk factors. Furthermore, the phenotype of CIAP resembles diabetic neuropathy both clinically and electrophysiologically. In sural nerve biopsies from patients with diabetes mellitus, structural abnormalities indicating microangiopathy in the endoneurial microvessels are well documented. Similarly, sural microvessel abnormalities have been shown in patients with atherosclerotic non-diabetic peripheral vascular disease. However, the reported histopathological alterations of microvasculature in sural nerves of CIAP patients are inconsistent. Two studies report microangiopathic changes in CIAP sural nerves comparable with those found in patients with diabetic neuropathy. Conversely, another recent study showed no significant differences in the microangiopathic parameters in the endoneurial microvessels in the sural nerve biopsies from CIAP patients compared to controls without polyneuropathy. However, this CIAP patient group was younger compared to the patient groups in the other two studies. A general limitation with the published morphological studies are that different methods have been used in the assessment of microangiopathy, and there is also a risk of subjectivity in the results. Immunohistochemistry studies of sural nerves with verification of microangiopathy using specific biomarkers would be of great interest to develop.

## Introduction

Polyneuropathy entails a diffuse dysfunction of the peripheral nerves. It is a common neurological disorder with an overall prevalence of 1.6% (1). The incidence of polyneuropathy increases with age (2), with a reported prevalence of 3.9–6.6% in the population older than 60 years (1, 3). Polyneuropathy is associated with impaired walking ability and an increased risk of falls (4). The most prevalent identifiable cause of polyneuropathy is diabetes (2, 5, 6). Other common etiologies are toxic, immune-mediated, vitamin B12 deficiency, and hereditary ones (2, 5, 6). However, about 25% of polyneuropathies remain idiopathic despite an extensive investigation (2, 6). A large proportion of these patients have a chronic idiopathic axonal polyneuropathy (CIAP), which is a slowly progressive predominantly sensory axonal polyneuropathy. The incidence of CIAP increases with age (2). Almost 50% of the middle-aged and elderly people diagnosed with polyneuropathy have CIAP (3), with a corresponding impairment of quality of life (7–10). The pathogenesis of CIAP is per definition unknown although there are several prevailing etiological hypotheses such as metabolic, vascular, and neurodegenerative causes. No disease-modifying treatment options are available in CIAP today (11).

This mini review addresses the association between CIAP and metabolic risk factors and will focus on the hypothesis of disturbed microcirculation in the microvessels (vasa nervorum) of peripheral nerves as a pathogenic cause of CIAP. We aim to provide an update of the present literature and the methodological limitations with the current studies and discuss a possible future area of development in the field.

## Association Between CIAP and Metabolic Risk Factors

Several research groups have demonstrated an association between different metabolic risk factors and CIAP (5, 12–22). In fact, just recently, Hanewinckel et al. showed that obesity and hypertension are associated with a decline in peripheral nerve function even before the emergence of polyneuropathy symptoms or signs (22).

The metabolic syndrome was found to be more common in Dutch patients with CIAP (55%) compared to controls (34%) and even more frequent in patients with a painful predominantly sensory CIAP (62%) (21). The risk factors implicated in the Dutch study were abdominal obesity and hypertension (21). A prospective Italian study presented an increased risk of developing CIAP in patients with peripheral vascular disease (20). Lipid abnormalities have been shown to be an independent risk factor for CIAP in some studies (5, 14, 16), but not in others (23).

Several studies [including one controlled study (13)] have designated impaired glucose tolerance as a strong risk factor for idiopathic sensory neuropathy (12, 13, 15, 18, 19, 24). However, this could not be confirmed in CIAP patients in a controlled study (14) nor has a high prevalence of large fiber neuropathy been identified among patients with impaired glycemic control (25). In a Swedish study, the authors found no difference in sural nerve conduction velocities between subjects with normoglycemia and those with impaired glucose tolerance (26).

## Microangiopathy in Sural Nerve Biopsies of Patients with Diabetic Neuropathy

Early qualitative studies of patients with diabetic neuropathy have described vascular abnormalities in endoneurial microvessels such as endothelial hyperplasia and obliteration of the vascular lumen (27, 28). Several histopathological studies of sural nerves from patients with diabetic (type I and II) diabetic distal symmetrical polyneuropathy (DSPN) have shown microangiopathic changes in endoneurial microvessels (29–40). However, the studies differ in the method of measurement and definition of microangiopathy, the type of controls used, whether the controls were age matched, and if the study had included only DSPN patients or even patients with diabetes without neuropathy. Table 1 summarizes the different microangiopathic parameters assessed and their corresponding significance in the controlled studies of DSPN patients.

**Table 1:** Overview of microangiopathic parameters assessed and reaching significance in the controlled studies of microangiopathy in diabetic distal symmetrical polyneuropathy patients.

References	Patients (*n*)	Controls (*n*)	BMA	MA	LA	EA	EN/V	EJ/V	PA	PC	PN/V	CC	CD
Dyck et al.^a^ (31)	36	45^c^	na	na	na	na	s*	na	na	na	na	s***	ns
Powell et al.^a^ (38)	11	7^d^	na	s***	ns^e^	na	na	na	na	na	na	na	na
Yasuda and Dyck^b^ (40)	34^f^	23^c^^,^^g^	s***	s***	ns	s***	s**	na	s***	ns	s***	na	na
Malik et al.^b^ (34)	21^f^	6^h^	s**^i^	s**^j^	s*^k^	s**^i^	s**	s***^l^	na	na	s**	na	s**^m^
Bradley et al.^b^ (29)	27	9^n^	s**	na	ns	s**	s**	na	na	na	ns	na	ns
Britland et al.^b^ (30)	16	6^n^	s****^o^	s****^i^	na	ns^i^	na	na	na	na	na	na	ns
Malik et al.^b^ (36)	15	8^h^	s****	na	ns	ns	ns	s**	na	na	s*	ns	s*^m^
Malik et al.^b^ (37)	20	10^h^	s****	na	s****	s**	na	s****	na	na	na	na	na
Giannini and Dyck^a^ (32)	54	50^c^	s^q^	na	ns	ns	ns	ns	s^q^	s^q^	s^q^	na	ns
Giannini and Dyck^b^ (33)	54^f^	50^c^	s**	s**	ns	na	s**	na	na	na	na	na	na
Malik et al.^b^ (35)	12	12^p^	s**	na	s**	ns	na	s**	na	na	ns	na	ns

*BMA, basement membrane area; CC, capillary closed; CD, capillary density; EA, endothelia area; EN/V, number endothelial nuclei/vessel; EJ/V, endothelial junction/vessel; LA, Lumen area; MA, mural area; na, non-applicable, i.e., not described in the article; ns, non-significant; PA, pericyte cell area; PC, periendothelial cell coverage; PN/V, number pericyte nuclei/vessel; s, significant*.

*p < 0.05, **p < 0.01, ***p < 0.001, ****p < 0.0001.

*^a^Diabetes patients regardless of neuropathy status vs controls*.

*^b^Diabetic neuropathy vs controls*.

*^c^Healthy subjects or volunteers*.

*^d^Five with normal morphology and two with nerve fiber loss but normal appearing vessels*.

*^e^Lumen diameter*.

*^f^Total number of included patients with diabetes both with and without neuropathy, the reported results in the table regards the patients with neuropathy*.

*^g^Not age-related controls*.

*^h^Brain-dead transplant donors and traumatic amputees*.

*^i^Thickness instead of area*.

*^j^Total vessel perimeter*.

*^k^Lumen perimeter*.

*^l^Endothelial cell profile number*.

*^m^Decreased capillary density in patients with diabetes with neuropathy*.

*^n^Organ donor cases*.

*^o^Basement membrane and pericyte cell thickness*.

*^p^Multiple organ donors, traumatic amputees and one patient with an osteosarcoma*.

*^q^p values not reported*.

The most consistent finding of microangiopathy yet reported is an increased basal membrane area or basal area thickness (29, 30, 32, 34–37, 39, 40). Other parameters indicative of microangiopathy are endothelial hyperplasia, i.e., increased number of endothelial nuclei per vessel (29, 31, 34, 40), increased endothelial profile number (34–37), endothelial hypertrophy, i.e., increased endothelial area (29, 34, 36, 40, 41), increased number of closed capillaries (31), decreased lumen area (35, 36), reduced endoneurial capillary density (34, 37), increased pericyte cell area (32, 40), and increased number of pericyte nuclei per vessel (32, 34). A recurrent qualitative description in DSPN patients is an increased reduplication of the basal laminae (29, 33, 40).

Microangiopathic changes are described in diabetic patients with both mild and subclinical neuropathy (33, 35, 37), and microvessel abnormalities are found to correlate with the severity of the neuropathy (31, 33, 34, 40).

## Microangiopathy in Sural Nerve Biopsies from Patients with Other Conditions than Diabetes Mellitus

### Chronic Ischemia Is Associated with Structural Abnormalities in Endoneurial Microvessels

Another indirect link between ischemia and CIAP consists of the reports of a higher frequency of polyneuropathy in patients with chronic obstructive pulmonary disease (COPD) and obstructive sleep apnea (42–46). However, in a large case–control study, the prevalence of COPD in CIAP patients was not higher compared to controls (47).

Stoebner et al. compared sural nerve biopsies from 13 patients with hypoxemic COPD with 9 age-matched controls and found electrophysiological abnormalities in all but 1 of the COPD patients although clinical signs of neuropathy were found only in 3 patients (48). Microangiopathic changes such as significant enlargement of the basement membrane, narrowing of the lumen, and increased mural pericytic debris deposits [also previously reported in DSPN patients (33)] were found in the COPD patients but not in the controls (48).

Similar changes have been shown in patients with atherosclerotic peripheral vascular disease without diabetes (49). McKenzie et al. showed increased endothelial cell area, as well as periendothelial cell area (including pericyte cells and basement membranes), and reduced lumen area in sural nerves from nine severely ischemic amputated legs in non-diabetic patients with chronic peripheral vascular disease and neuropathy compared to four controls (49).

### Microangiopathic Changes in Hereditary Neuropathy: A Secondary Phenomenon?

In a search for sural nerve microangiopathy in 27 DSPN patients, Bradley et al. used 9 hereditary sensory motor neuropathy (HMSN) I patients as a control group, since the neuropathy in HMSN I has no known vascular pathogenesis (29). A control group of nine organ donors with no known neuropathy or comorbidity associated with neuropathy was also used. Surprisingly, the HMSN I patients had a significantly larger endothelial cell area than both DSPN patients and controls. Both the DSPN and HMSN I patients had increased number of endothelial cell nuclei per vessel compared to controls. The HMSN I patients had a significantly larger basal membrane area compared to controls. These endothelial findings in HMSN I patients suggest microangiopathy to be secondary to the neuropathy, rather than causative for it.

## Histopathological Studies of Sural Nerves in Patients with CIAP

Considering the clinical and electrophysiological phenotype resemblances of CIAP and DSPN (50), one would expect comparable microangiopathic findings in endoneurial microvessels in patients with CIAP as in DSPN patients. The question regarding microangiopathy in CIAP patients has been explored in three morphological studies (50–52).

### Primary Results

In year 2000, Teunissen et al. found significantly larger basal lamina area thickness (BLAT) in sural nerves of Dutch CIAP patients (*n* = 18) in comparison with HMSN II (*n* = 6), but not compared to autopsy controls (*n* = 10) (*p* = 0.08) (52). The endothelial cell area was significantly higher in CIAP compared to HMSN II, but lower compared to autopsy cases probably due to postmortem changes. The BLAT and the endothelial cell area in CIAP (*n* = 18) and DSPN patients (*n* = 4) were in the same range. Regarding the number of endothelial cell nuclei, there was no difference between CIAP and HMSN II and autopsy controls. There was no significant difference in lumen area, number of pericyte nuclei, or capillary density between groups. In accordance with earlier reports (29, 33), Teunissen et al. found reduplication in basal laminae in patients with DSPN, but not in CIAP patients (52).

Moreover, Teunissen et al. investigated the frequency of peripheral arterial disease in their group of CIAP patients (52). Seven of the 18 CIAP patients in their study had abnormal ankle brachial index. BLAT was enlarged in CIAP patients with a low ankle brachial index *p* < 0.01, but was similar in patients with and without manifest vascular disease, defined as the prevalence of ischemic cardiac disease or stroke. Unfortunately, Teunissen et al. did not report the subgroup analysis comparing BLAT in CIAP patients with normal ankle brachial index, with BLAT of HMSN II patients and autopsy cases (52). This would have been of interest, since one cannot exclude the observed enlargement of BLAT in the CIAP group to have been influenced by the peripheral arterial disease, as has been reported by others (48).

More recently, two other studies assessing microangiopathy in sural nerves of CIAP patients have been published (50, 51). Samuelsson et al. compared sural nerve biopsies from 10 Swedish CIAP patients with 11 cases of inflammatory neuropathy and 10 biopsies from subjects without sensory polyneuropathy (51). They found no significance in any of the microangiopathic parameters in CIAP patients compared to the controls without neuropathy. The BLAT and the endothelial cell area were significantly higher in the group with inflammatory neuropathy than in both the patients with CIAP and the controls without polyneuropathy. In subgroup analysis, these results were shown to be restricted to the subgroup of vasculitic neuropathy and not to patients with chronic inflammatory demyelinating polyneuropathy (CIDP).

Hube et al. have recently published a study of 30 German patients with CIAP, 28 with DSPN and 31 healthy controls (50). No overrepresentation of the metabolic syndrome was detected in CIAP patients compared to healthy controls (50), hence not confirming the results from other studies (5, 20, 21). In the assessment of individual parameters from the metabolic syndrome, hypercholesterolemia was more prevalent in CIAP than in controls (50). The basement membrane thickness and the number of endothelial cell nuclei in the endoneurial microvessels of the 10 CIAP and 7 DSPN German patients who had undergone a sural nerve biopsy did not differ significantly leading the authors to conclude that microangiopathy was present in CIAP patients.

### Differences in Patient Characteristics between the Three Morphological Studies

#### Age and Gender

The mean age in the predominantly male (83.3% males) Dutch CIAP group was 63.0 years (52). In the 10 Swedish CIAP patients with an even gender distribution, the mean age was 54.9 years, with a median age of 57 years and a range of 25–78 years (51). In the German study with an even gender distribution (53% males), the mean age reported for the CIAP patient group was 61.1 years (50). However, this was the mean age and gender distribution for the whole group of 30 CIAP patients and not only the subgroup that had undergone a sural nerve biopsy (50).

It is possible that the discrepancy in the three studies regarding increased BLAT in CIAP patients (50–52) can partly be explained by the younger group of CIAP patients studied in the negative study (51).

##### Is There a Correlation between BLAT and Age?

The results of studies examining the relationship between BLAT and age are somewhat inconsistent although the studies reporting no age relation outweighs (33, 40, 51–54). Yasuda and Dyck describe no relation between basal membrane thickness and age in young healthy subjects (mean age, 30.9 years; range, 20–54 years) (40). Giannini and Dyck found no significant correlation between the basement membrane area and age in 53 healthy volunteers, 22–66 years of age (53). In CIAP patients, no correlation between age and BLAT was found (33, 52). However, Samuelsson et al. identified a positive correlation between age and BLAT in the whole cohort of patients and controls (51). Jacobs and Love have reported prominent reduplication of the endothelial and pericytic basement membranes (but not the membrane itself) of the vasa nervorum from the sixth decade and onward in 27 postmortem sural nerve biopsies in the age range of 0–77 years (54).

### Differences with Regards to the Control Groups between the Three Morphological Studies

In the Dutch study, three different age-matched control groups (DSPN *n* = 4, HMSN II *n* = 6, and autopsy cases *n* = 10) were examined. DSPN was chosen as a positive control due to the known association of DSPN with endoneurial microvessel abnormalities (52). HMSN II patients were chosen as a negative control since vascular pathogenesis is deemed unlikely in hereditary neuropathies. The sural nerve autopsy biopsies were extracted within 24 h (mean, 11.5 h) postmortem. Medical records were reviewed, and patients with a possible polyneuropathy or a comorbid disease such as diabetes mellitus known to cause a polyneuropathy were excluded.

The controls in the Swedish study were age and gender matched and consisted of 2 groups: one with 11 patients with inflammatory neuropathy (CIDP = 6, and vasculitic neuropathy = 5) and one group with 10 controls given other diagnoses than sensory polyneuropathy on follow-up examination (51). The two groups of inflammatory controls differed regarding the primary site of inflammation, i.e., myelin in CIDP vs epineurial and endoneurial blood vessels in vasculitic neuropathy. This is reflected in the subgroup analysis where the CIDP group had much less vascular abnormalities than the patients with vasculitic neuropathy.

In contrast to the other two above-mentioned studies (51, 52), Hube et al. had no normal control group for the biopsy section of the study (50), but rather used 7 DSPN patients as positive controls for their 10 CIAP patients.

### Differences in Study Design and Methods Used in the Three Studies

The Dutch study was a prospective study (52), unlike the latter two retrospective ones (50, 51).

Electron micrographs from ultrathin EM sections were used to measure the microangiopathic parameters in the Dutch and Swedish study (51, 52). They used the same mathematic calculation to assess BLAT as their main microangiopathic parameter, i.e., BLAT was calculated by subtraction of the radius of a circle equivalent of lumen and endothelial area (LEA), from the radius of a circle equivalent of the total vessel area (VA) (BLAT = √VA/π − √LEA/π). In the German study, the basement membrane thickness of each endoneurial microvessels was evaluated by viewing the specimens in semi-thin sections, i.e., by light microscopy (50).

## Limitations with the Present Studies

The three studies exploring microangiopathy in the endoneurial microvessels in CIAP patients differ in design, method to assessing microangiopathy, and the nature of the control groups (50–52). They are all small studies with regards to the number of patients and controls (50–52). The retrospective set up is limited due to a selection bias where the final diagnosis is determined by the neuropathological evaluation of the sural biopsy (50, 51). The basement membrane of the endoneurial microvessels are multiple and fragmented, which implies difficulties to measure the thickness and the total length of the basement membrane (53), which was the method used in the German study (50). A mathematic calculation of BLAT used in the other studies diminishes this constrain (51, 52). However, there inescapably seems to be a significant limitation in the morphological methods used to measure microangiopathy in sural nerves in these previous studies, mainly since they all include a certain extent of subjectivity in the assessment.

A further limitation is the variance of the control groups. The German study lacked a normal control group (50). Autopsy cases indicate a risk for postmortem changes, probably reflected as a postmortem increase in endothelial area (52). The normal controls used in the Swedish study had symptoms that had prompted the biopsy, so despite not having a sensory neuropathy affecting the sural nerve, they were not completely healthy (51). Also the selection of the patient group can be questioned. In the Dutch study, the results can have been affected by the co-existing peripheral arterial disease diagnosed in the CIAP group (52). The Swedish CIAP patients with a mean age of 54.9 years and a range of 25–78 years (51) were younger than most CIAP patients whose clinical symptoms usually do not start until the 6th decade (55).

The basal membrane area or thickness is often highlighted as the most prominent and consistent microangiopathic marker of ischemia in peripheral nerves. However, increased basal membrane area has been shown even in HMSN I patients, where vascular pathology is not a part of the pathogenesis of the neuropathy (29). The authors suggest the increased basal membrane area to be secondary to the neuropathy and not the cause of it (29). However, later studies have shown that basal membrane thickness precedes DSPN (33, 35, 37) and that repeated axonal degeneration and regeneration in rat nerves does not increase endoneurial basal membrane reduplication in microvessels (56), thus arguing against the speculation that microvessel changes may be secondary to the neuropathy.

So in summary, the major limitation with the present mini review is the small number of available reviewable studies dealing with the process of microangiopathy in peripheral nerves in CIAP, making it difficult to arrive at any definite conclusions.

## Further Perspective

To perform a prospective sural nerve biopsy study with a healthy control group would be impossible to perform at least in Sweden today due to ethical regulations. Sural nerve biopsy is not a part of the regular etiological clinical investigation in CIAP patients. The sural nerve biopsy is an invasive procedure with a risk for longstanding sensory impairment and neuropathic pain in the innervated area of the sural nerve. So the retrospective set up and the selection bias with the final clinical diagnosis determined by the neuropathologist will probably have to be used even in future biopsy studies.

The question is rather how to avoid the subjectivity and to find a more specific histopathological marker representative for microangiopathy. The increased basement membrane area in CIAP patients is reported to consist mainly of collagen in contrast to DSPN patients in whom the lamina reduplication is probably the main the cause of the increase in BLAT (52). Immunohistochemistry study of collagen may be a possible way to further explore this issue.

## Conclusion

It remains unresolved whether microangiopathy actually exists in endoneurial microvessels in CIAP, since the results of the small and methodologically limited studies exploring histopathology in sural nerves of CIAP patients are inconsistent. Furthermore, if microangiopathy does exist, it may in part be caused by ischemia due to co-existing peripheral arterial disease.

Immunohistochemistry studies of sural nerves with verification of microangiopathy using specific biomarkers may aid in resolving the uncertainty whether CIAP is associated with small vessel disease in the peripheral nervous system.

## Author Contributions

KS and RP were involved in the design and intellectual concept of the study. KS performed the literature search. KS drafted the manuscript. RP critically revised the manuscript. KS and RP finally approved the work and agreed to be accountable for all aspects of the work in ensuring that questions related to the accuracy or integrity of any part of the work are appropriately investigated and resolved.

## Conflict of Interest Statement

The authors declare that the research was conducted in the absence of any commercial or financial relationships that could be construed as a potential conflict of interest.

## Abbreviations

BLAT, basal lamina area thickness; CIAP, chronic idiopathic axonal polyneuropathy; CIDP, chronic inflammatory demyelinating polyneuropathy; COPD, chronic obstructive pulmonary disease; DSPN, diabetic distal symmetrical polyneuropathy; HMSN, hereditary motor sensory neuropathy.
